# Identities Hidden in Challenges: The Sequential Mediation of Thriving at Work and Employee Investment

**DOI:** 10.3389/fpsyg.2020.555420

**Published:** 2020-11-20

**Authors:** Sharjeel Saleem, Shazia Humayun, Bilal Latif, Umer Iftikhar, Imran Sharif

**Affiliations:** ^1^Lyallpur Business School, Government College University Faisalabad, Faisalabad, Pakistan; ^2^Department of Leadership & Management Studies, National Defence University, Islamabad, Pakistan; ^3^Lahore Business School, The University of Lahore, Lahore, Pakistan

**Keywords:** challenge stressors, thriving at work, employee investment, identity orientation, sequential mediation

## Abstract

The present study explores the influence of challenge stressors on identity orientation directly and via thriving at work and employee investment. Drawing on the broaden–and–build theory of positive emotions, this study proposes challenge stressors as a critical predictor of identity orientation. The purpose of this article is to explore if a particular identity is salient in different contextual factors, and this study suggests that challenge stressors stimulate personal, relational, and collective identities to respond to a situation. The relationships hypothesized in this study were tested using a sample of 225 employees from the banking sector of Pakistan. A time-lagged research design consisting of two waves of data collection was employed. A structural equation modeling technique was used to test the hypotheses regarding the relationship between challenge stressors and identity orientation, including the role of thriving at work and employee investment as intervening mechanisms of this relationship. Results showed that challenge stressors had a significant positive relationship with identity orientation. The results also confirmed the sequential mediation of thriving at work and employee investment in the relationship between challenge stressors and identity orientation. The findings suggest that the positive side of stress as a strength motivates employees for continued self-development. Importantly, challenge stressors enhance employees’ ability to thrive at work and, in turn, they invest in the work more and identify themselves strongly with their organization and work.

## Introduction

Identity is comprised of interpersonal and intrapersonal relationships, actions, and behavioral adjustments in response to adjacent contextual factors. The self-identification of high-potential talent is the cornerstone for an organization’s growth and competitive advantage (Frone et al., 1995; Berzonsky and Ferrari, 1996; Sturman et al., 2003; Tjosvold et al., 2003; Haslam et al., 2004; Thatcher and Zhu, 2006; Jackson et al., 2007). The self-identification of talented employees not only contributes to macro-level outcomes but also positively shapes other organizational members’ attitudes and behaviors. Relational and collective identities are socially construed and are context-bound, highlighting that the behavior of people is more reliant on group members’ attitudes, affiliations, and external environment (Cheek and Cheek, 2018). Intrinsic motivation is the driving force in personal identity, which makes individuals more empowered, self-reliant and productive in a given situation. The literature on the outcomes of identity, such as self-identity (Kira, 2019), relational and social identity (Haslam et al., 2004; Valaei and Rezaei, 2016; Evans et al., 2019), organizational identity (Gonzalez and Chakraborty, 2012), physical identity, and work and role identity is abundant (Brickson, 2000). “What are the major root causes which stimulate the particular type of identity?” is still a pertinent question. This research question also highlighted the underlying mechanisms which relate challenge stressors to identity orientation. Diverse values, beliefs, and styles reside in each person, but the variance lies in just how a particular identity is initiated and why it is initiated is still a gap in the literature which we try to answer in this study.

The literature on occupational stress spans several decades (Beehr and Franz, 1987). It has remained a relevant area of research in management and psychology as organizations have to bear losses amounting to millions of dollars in the shape of stress-related costs, e.g., loss in productivity and medical costs (Cynkar, 2007). Most of the research has focused on the negative side of the stress; however, research has revealed that certain aspects of stress may also have a positive impact on employees as well as organizational outcomes (Podsakoff et al., 2007; Wallace et al., 2009). According to this discussion, job stressors are of two types: hindrance and challenge stressors. Challenge stressors refer to “work-related demands or circumstances that, although potentially stressful, have associated potential gains for individuals.” Hindrance stressors refer to “work-related demands or circumstances that tend to constrain or interfere with an individual’s work achievement and that do not tend to be associated with potential gains for the individual” (Cavanaugh et al., 2000, p. 68).

Coping strategies of stress like stress as a challenge is a powerful tool, which intrinsically motivates employees to feel empowered, autonomous, and self-guided, and they thrive at work to get rid of stressful situations. Likewise, for social identification, self-enhancement and self-esteem are the most important motivators (Sluss and Ashforth, 2007). Studies on identities emphasize how group participants feel about, in what way the members treat them, and by what means they build their association within the group (Hashimoto and Kudo, 2010; Cheek and Cheek, 2018). In this study, we argue that challenge stressors can be a predictor and situational factor which incites the level of identity in an individual.

The transactional theory of stress and coping, developed by Lazarus and Folkman (1984), states that employees face positive and negative stress, which leads to the evaluation of tasks as either challenge (positive) or hindrance (negative). This theory explains how employees respond to stressful situations. Stress at the workplace has received considerable scholarly attention in recent years, regarding its concerns both for individual employees and groups (Crane et al., 2018; Kira, 2019).

The current study has two main objectives. First, this study is conducted to enhance empirical research to present a theoretical framework by linking job-related stress and employee identity orientations. Second, this study provides a plausible explanation of the mechanism of thriving at work and employee investment, and how employees are motivated and give their time and potential to the organization and achieve self-esteem and identity. The apparent theoretical links among challenge stressors, stress-related problems, and thriving at work, call for further exploration. Spreitzer et al. (2005) defined thriving as being invigorated and enthusiastic, being passionate, the sentiment and sensation of being esteemed, and the sensation of being appreciated. Employee investment is expressed as a credit conferred on employees in return for one’s investment of energies in the organization in a particular time period (He et al., 2014).

To date, researchers have mostly emphasized the direct effects of organizational stress on employee health, attitude, well-being and their conducts (Park, 1998; Hardie, 2005; Widmer et al., 2012; Berjot et al., 2013). We rationalized the need to comprehend the stress with the motivation of employees. Mainly the motivation behind this research is to propose the relationship of challenge stressors and identity. So we assume in this study that the significance of employee performance is contingent on the employee’s motivation. The main purpose of this investigation is to determine the sequential mediating impact of thriving at work and employee investment in the relationship between challenge stressors and identity orientation. We argue that challenge stressors, being the positive side of stress, is a strength that motivates employees to achieve continued self-development and invest in developing relationships with the organization, which leads to personal, relational and collective identity orientation. Coping with the challenge stressors is a source of motivation and in doing so, employees get an opportunity for self-growth and learning (Prem et al., 2017), and learning is an essential part of thriving at work. Numerous studies have found a positive impact of thriving at work on organizational commitment (e.g., Porath et al., 2012). In the same vein, we argue that employees who thrive at work, and are committed to the organization, would invest their personal resources to deliver an extraordinary performance. When employees have their goals and values aligned with those of the organization, and their KSAs are in congruence with the requirements of the job, they identify with the organization (Kasimati, 2011).

This study offers inferences for improving member credentials within the organization, which relates to increased contribution and continual membership. A positive external appearance increases the probability that organizational associates have values, principles, and interests, which lead to the establishment of leaders and stable followers (Podsakoff, 2007). The notion of identity described here represents identity as a multidimensional construct by emphasizing three separate dimensions of identity orientation (i.e., personal, relational, and collective identity). Either the person perceives himself as an individual character or in relation to group members within the organization; it, too, influences the means by which a person assesses himself in personal and relational identities.

This study makes numerous important contributions. First, it investigates the unexplored link between challenge stressors and different aspects of identity orientation and relates it to employee behavior. To the best of our knowledge, our study is the first study that explores this link. Second, drawing on the broaden–and–build theory (Fredrickson, 2001), the current study augments the emergent literature on identity orientation by justifying challenge stressors as its antecedent. Theoretically, in the previous literature, the concept of challenge stressors has been widely supported by transactional stress theory (Salanova et al., 2011; Amah, 2014; Stiglbauer and Zuber, 2018) and the conservation of resource theory (Hobfoll, 1989, 2012; Hobfoll et al., 2012, 2018). However, the mechanism which relates challenge stressors to the particular identity is still under-discussed. In this study, we have used a nuanced theoretical approach based on the overarching framework of broaden–and–build theory, to understand the phenomenon which stimulates positive identity. Third, we also suggest a serial mediational mechanism comprised of thriving at work and employee investment as mediators that link challenge stressors to the identity orientation. In previous literature, thriving at work has been linked with challenge stressors (e.g., Prem et al., 2017). However, thriving and employee investment have not been examined as mediators between challenge stressors and identity orientation. Fourth, this study is conducted in the commercial banking sector of Pakistan which is characterized as a collectivistic society which, thus, makes Pakistan an exciting study setting as it is interesting to see how collective orientation is impacted by challenge stressors in a collectivistic culture.

For this study, the data were gathered from the banking industry. Banking sectors play a significant role in upgrading the economic structure of any country. Financial sector reforms are the ultimate source to improve the structure of the banking sector; resultantly commercial banks are finally able to capture maximum market share. The commercial banking sector in Pakistan has seen tremendous growth in the last couple of decades. This growth, however, has brought a lot of stress and challenges for the employees. The employees in commercial banks of Pakistan ought to meet the challenging and, oftentimes, unrealistic targets. Keeping in view the theoretical underpinnings of the study, the target population is relevant as these employees face stress very often. This competition brings about a lot of pressure to compete, learning challenges and work-related stress. To study the challenges related to employees of the banking sector and how they deal with stress is the basic agenda of this study.

## Theory and Hypotheses

### The Broaden–and–Build Theory as an Overarching Framework

The broaden–and–build theory of positive emotions (Fredrickson, 2001) proposes that “experiences of positive emotions broaden people’s momentary thought action repertoires, which in turn serves to build their enduring personal resources, ranging from physical and intellectual resources to social and psychological resources” (Fredrickson, 2001, p. 218). Similarly, positive emotions help to mend personal resources like skills, abilities, and social networking, which lead to self-fulfillment and well-being (Rodell and Judge, 2009). When individuals feel accompanied by sufficient resources, it enables their judgment to perceive the situation from a different perspective. It is believed that the broaden–and–build theory enforces positive emotions which ultimately expand the person’s psychological thinking patterns and actions (Fredrickson, 2013). The person critically evaluates the problems and finds multiple means to get rid of the stressful situation. In the first phase, an individual broadens one’s cognition and gets a clue from the external environment, adopts the coping strategies of stress, and attaches it to the future benefit (Sun et al., 2020).

The target population for this study was employees from the commercial banking sector of Pakistan. We cannot separate the banking sector from stressful situations; the fear of falling short of business targets, keeping the customers satisfied, and enhanced learning demands induce continuous pressure and stress among the employees of the banking sector. Handling of stressful situations in a challenging way allures the feeling of pleasure, which helps to manage the conflicting results of negative emotions. This process initiates the second phase and builds enduring intellectual competencies which, in turn, support resilience, enthusiasm, physical health, and well-being (Spreitzer et al., 2005). Building personal resources not only secures one’s image but also enhances supportive activities in the organization. This makes employees confident to accept challenging tasks and take calculated risks, which contributes to thriving and self-development; and, in turn, broadens the extra-role behaviors (Mikulincer and Shaver, 2019).

Work is an imperative domain, and an individual wishes to create positive identity in this zone for self-admiration and to gage meanings in life. The purpose is to acknowledge themselves by defining in a particular way. This article attempts to study the unexplored link between challenge stressors and positive identity leading to the explanation of an individual’s adaptation in organizations (Dutton et al., 2010). In doing so, we study thriving at work and employee investment as mediators, which is another novel contribution of our study. According to the broaden–and–build theory, people who like to be involved in relationships and interactions with each other are more likely to engage themselves in innovative roles and prosocial behavior (Coyle-Shapiro and Conway, 2005). Self- enhancement is the primary driver of identification. Commonly people prefer to be part of a group that has high social values and recognition. Drawing on the broaden–and–build theory, this study argues that taking the positive side of stress as a strength motivates employees to achieve continued self-development and invest in developing relationships with the organization, leading to the development of high social standard communities. One’s feeling of strong affiliation with a distinctive group may be uplifted by positive emotions due to coping with stress at work. His awe emotions motivate him to overcome stressing situations and to engage actively in productive tasks which broaden his expertise for future ties. The degree to which a person perceives himself as de-individualized, the more they invest in relationships leading to the strong feelings of satisfaction stemming from being a part of the community (Cai and Chau, 2016). Their activities are based on collective goals, and social interactions, which provoke the status of well-being.

### The Relationship Between Challenge Stressors and Identity Orientation

It is perhaps not uncommon to witness work-related stress as it is a primary concern for managers to pinpoint the impact of this phenomenon on the workforce. According to the Harvard and Stanford researchers, in the United States each year, $190 Billon health-related costs are incurred, and 120,000 deaths of employees take place due to workplace stress (Davenport, 2015). The research on stress at work had passed through many phases from the time when it was firstly announced as workplace stress (Sharp, 1996; Cavanaugh et al., 1998; Haslam and Reicher, 2006; Berjot et al., 2013; Amah, 2014). The earlier researchers defined acceptance of challenge as a way to ensure effectiveness within the workplace, which leads to an emotional state of accomplishment or self-actualization, resulting in positive outcomes (e.g., Miquelon and Vallerand, 2006; Amah, 2014). Commonly, challenge stressors are linked with vigilant or vigorous systems of managing; they raise one’s spirits, tactic, ingeniousness, and problem-focused approach (Gomez-Lanier, 2018).

Cavanaugh et al. (1998) introduced that work stressors could be distinguished in two types: those which have a tendency to be perceived as developmental and promoting individual growth (challenge stressors), and those which are perceived as a burden or obstacle to assignment completion and individual growth (hindrance stressors). Commonly, field reviews (Webster et al., 2010) plus meta-analytic assessments (Podsakoff et al., 2007) had revealed that this approach could be used to describe unpredictable connections among stress and worker approaches, opinions, behaviors, and performance. Huang et al. (2018) explored the role of challenge stressors as a mediator between high-performance work systems (HPWS) and employee engagement, which showed the direct linkage of both. Giorgi et al. (2020), in their study on expatriate employees’ cross-cultural adjustment, found the degree of adjustment to be negatively related to work-related stress.

Nevertheless, the following four dimensions of stress may be taken by the employee as a challenge: workload is the first dimension of challenge stressors. Spector and Jex (1998) argue about the quantifiable assignment, which refers to the quantity and volume of job work which is compulsory for individual’s title role within the group. Workload stress would be supposed as challenging since it involves completion of responsibilities which are essential for an individual’s role, and it helps to illuminate their identity inside the working organization. Work pace is the second task demand that would likely be considered as challenging to the workforce; it can be defined as the speed or relative rate in which tasks should be finalized to fulfill one’s job requirements. Latham and Locke (1991) have distinguished that in specific situations, challenging objectives motivate personnel to achieve their goals at advanced levels in which workers express their full potential. Job complexity is the third role demand that would likely be evaluated as a challenge in the place of work. According to Hackman and Oldham (1980) job characteristics theory, variety or breadth of role-related actions which are performed by the workforce in the organization is known as job complexity. In this way, complex tasks involve a wide variety of roles than less composite jobs. Job responsibility is the fourth sort of role demand that must be observed as challenging in organizational work. This role demand is linked with challenge evaluations because it deals with intrinsic and extrinsic obligations associated with development and expansion in the group.

Regardless of the series of literature that incorporates the relationship of challenge stress to social identity (Haslam et al., 2004; Haslam and Reicher, 2006; Webster et al., 2010; Amah, 2014), only a small number of scholars geared up to apprehend the fundamental mechanisms that linked the challenge stressors to personal and relational identity. Cruwys et al. (2014) revealed that a better level of social identification along with physical and emotional well-being is reached due to challenge stress. Stiglbauer and Zuber (2018) emphasized that management of stress is essential for organizations as well as for individuals, which helps to moderate the consequences of stress by assembling capabilities at a personal level. This finding is also discussed by Wang et al. (2017), which strengthens the evidence that challenge stressors are imperative to formulate different identities. Drawing on the broaden–and–build theory, the poised model contributes to the existing literature by investigating the direct relation of challenge stressors to personal, relational and collective identities and the mechanisms which explain this relationship.

More precisely, personnel who come across a lot of work pressure, and perceive that their job is not as much of importance, identify rarer opportunities for expansion and innovation, and do not exert efforts toward self-development. Drawing on the broaden–and–build theory of positive emotions; however, we argue that workers who manage work demands in the shape of contentment toward their role tasks, are loaded with positive emotions. Positive emotions gathered by meeting the challenging work demands serve to enhance resilience. The positive emotions, so gathered, widen the scope of attention and cognition and serve to boost individuals’ coping resources (Aspinwall, 1998, 2001; Fredrickson and Joiner, 2002). They have a more positive time in the workplace, have more chances of growth, and stronger insights toward self-worth, self-respect, and self-development (Seery, 2011). An employee’s ability to cognitive restructuring to perceive stress as a challenge is a fundamental tool to lessen feelings of depression (Garland et al., 2011). This would justify that a person’s job-fit may define one’s personal identity, which is noteworthy for their work-life balance. Previous research shows that identity orientation is linked with several cognitive aspects like the novelty of ideas, self-esteem, and self-recognition (Vignoles et al., 2006).

In social identity theory, the term “salience” is used to indicate the initiation of a positive identity (Tajfel et al., 1979). An investigation about the salience of social identity suggests different phenomena that determine why people are distinct regarding individual and social identity. Identity orientation literature focuses on how one’s own self is defined concerning others. Three different identity orientations, personal, relational, and collective, can be differentiated on the basis of motivation (according to perceived values), nature of appropriate self-knowledge (how they like to define them?) and the frame of reference, which evaluates them. Primarily, the concept of identity orientation points out “who am I?” and also “How should I act?” (Brewer and Gardner, 1996). It accentuates strictly how individual description of identity might be influenced by social relations and the resultant chemistry of three “stages” of identity: individual (personal), relational, and collective (group, social). Conferring to above discussion and other researchers (e.g., Flynn, 2005; Vignoles et al., 2006; Evans et al., 2019), it can be elucidated that the personal identity relies on one’s perception of being unique, interpersonal assessments of individualities, capabilities, commitments, and enactment, which create self-esteem. The adoption of social identity is motivated by self, the need for connectedness, interdependence, self-improvement, and evaluation of positive identity. This study depicts a clear vision of how people describe and find themselves within organizational settings.

Preliminary empirical research deepens the linkages of employee self-identity with commitment, regulatory focus, and citizenship behavior (Evans et al., 2019). Haslam et al. (2004) investigated the relationship between stress and social identities. According to them, in-group members trust the information which they get from the same group members. The information from the in-group person that situations are stressful has more persuasive meaning and becomes more of a challenge, than the same information provided by the out-group person as members carried the indistinguishable societal perception. So, in light of the above-presented literature and arguments, we propose the following hypothesis:

***H1.*** *There is a positive association between challenge stressors and identity orientation.*

### Thriving at Work and Employee Investment as Mediators

Thriving at work explains the feelings of association and connectedness with people, associating positive feelings with actions taken. Thus, thriving is about being productive and focusing on competencies to acquire new ideas. So we conclude that eagerness to learn as well as to grow is known as thriving. A socially embedded model of thriving at work proposed by Spreitzer et al. (2005) defined thriving as a sense of progress in one’s self-development and holdup the progress of negative feelings (van der Walt, 2018). From this explanation, thriving can be defined as the reaction of challenge (for the reason that thriving denotes gain), not only to merely come back from pressure but also to respond to the challenging situations. These ideas describe thriving as a dual understanding of learning and vitality. According to Spreitzer et al. (2005), learning and vitality are vital constituents of thriving. If a person has ample learning but has sensations of burnout and exhaustion, then it cannot be supposed as thriving. While thriving at work is considered as a personal sense of flourishing, a person must be passionate about work (Stiver and Miller, 1998) and have self-confidence and proficiency (Wallace et al., 2016). On the contrary, if an individual is invigorated and thrilled but feels their ability to learn as ordinary, the same individual may not be in the state of thriving. Therefore, these two measures include both dimensions of psychological involvement, i.e., the cognitive (learning) and emotional (vitality) correspondingly. Furthermore, the description of thriving by means of growing in expressions of both vitality and learning take likewise the eudemonic (learning) and hedonic (vitality) characteristics of psychological development (Waterman, 1993).

### The Relationship Between Challenge Stressors and Thriving at Work

As described above, in this article, the broaden–and–build theory of positive emotions is an overarching theory which supports the fundamental framework. According to this theory, positive moods and emotions set a stage for more challenging behavior which is imperative to cope with stress. Challenge stressors link worker’s emotions and capabilities with achievement, and these rising signs propose the significant relations with enthusiasm, commitment, task performance, and employee well-being (Norlander et al., 2005; Jackson et al., 2007; Levene, 2015; Prem et al., 2017; Zhai et al., 2017; Eustis et al., 2018; McNeill et al., 2018). According to the broaden–and–build theory, positive emotions help to build personal resources. Thriving consists of energetic and deliberate commitment (Prem et al., 2017) in the course of personal development. Prem et al. (2017) further explored the impact of personal resources, learning demand and time pressure (challenge stressors) on learning and vitality mediated through the cognitive appraisal. Each dimension of thriving would contribute toward growth, particularly one’s self-development. If a person, however, feels exhausted, thriving suffers. We propose thriving at work as an intervening mechanism which relates challenge stressors to identity orientation through its mediating role. So we draw our next hypothesis as follows:

***H2.*** *There is a positive association between challenge stressors and thriving at work.*

### The Relationship Between Thriving at Work and Employee Investment

Spreitzer and Sutcliffe (2007) pointed out that persons who are active within organizations are overwhelmed with most of the employment responsibilities, as well as being expected to have new ideas and have more opportunities to place these ideas into the completion of tasks. Similarly, people who practice additional learning during the job (second dimension of thriving) are expected to be more competent and to acquire massive learning for improvement in organizational enactment. Fostering a continuous learning culture is a vital postulate of the broaden–and–build theory. Building a learning-intensive culture and a secure environment where employees are encouraged to work on innovative and novel ideas allows an organization to nurture the positive emotions in the minds of the employees (Lino, 2020). Learning might consist of new abilities, skills, and expertise which induce how to perform more effectively at the prescribed job role (Spreitzer et al., 2005). Learning may be shared directly among group members to yield more learning in an organization which may induce relational identity. When workers at the workplace feels passionate and are motivated by intrinsic and extrinsic rewards, they think of themselves like the owner of the organization and invest in their skills and potential for the progress of organization. This urge to do work and perform more efficiently motivates the employee to invest positively in the organization (Carmeli and Spreitzer, 2009). Challenge stressors raise enthusiasm which drives employee engagement (Bakker et al., 2016; Bakker, 2018; Huang et al., 2018; Bowler et al., 2019) and commitment (e.g., Abid et al., 2019); so we propose here that:

***H3.*** *There is a positive association between thriving at work and employee investment.*

### The Relationship Between Employee Investment and Identity Orientation

Usually, employee investment consists of definite work/job-related investment, training, and development in building personal relationships with coworkers and supervisors and socializing in the environment and ethos of the organization. As defined by Farrell and Rusbult (1981), “Investments refer to the resources that are ‘put into’ an association, usually, but not necessarily, with the intent to improve the long-term value of the relationship. Length of service, acquisition of non-portable skills, and retirement programs are common job investments” (pp. 81–82). Research determined the fact that investment in the work is a firm footing of behavioral deviations from negative feelings to a positive outcome (Cohn and Fredrickson, 2006). By investing in their roles, organizational members show commitment and association with the organization which induces multiple identities. Literature attempting to clarify the behavioral perspective of employees argues that investment in a role is driven by pleasure-seeking considerations, mostly they invest their time and efforts in roles that provide pleasant feelings and satisfaction (Rothbard and Edwards, 2003). We, here, provide a plausible explanation about one’s identity seeking approach which leads to investing in role tasks. We propose a new stream of thoughts by highlighting that investment in the role is directly linked with the establishment of identification with the organization which explains why a person prefers one role over the other which motivates a particular level of identity and supports his bonding with the organization. Thus, the relationship between employee investment and identities is still a gap in the literature and needs to be further explored. Maskell et al. (2017) described investment in individuals as one of the critical performance-related constructs for the progress of the organization. Rodrigues and Lopes (2013) explicated that investment and cost related to employee progress may perhaps be interpreted into measurable organizational identities.

The broaden–and–build theory framework denotes the building of personal resources as employees’ investment such as an individual’s psychological and cognitive efforts and abilities that have been placed in a relationship (e.g., companionship, marital, occupation, and organizational). Drawing on the broaden–and–build theory framework, which states the notion of investment as a combination of skills, capabilities, and personal resources, which an employee invests in relationships; we argue that these ties and bonding make stronger footing in an organization which magnifies the cost of terminating the relationship. Job/role appears to attain an essential position gradually in the minds of people, often which explains their specialized capabilities or certified relationships. It would be considered as an imperative source of identification, assisting in defining particular individual and organizational identities (Alvesson, 2000, 2010). In the light of the investment model, a person’s administrative role (power of identification by way of contribution in a specific organizational job) rests not merely on the costs and rewards which linked work or work-related substitutes, but also depends on the degree of the person’s investment/contribution in the organizational job (Farrell and Rusbult, 1981). The study by Wilson et al. (2016) depicts the strong relationship between professional identity and commitment. According to their research, a strong sense of professional identity led to an investment of skills which resultantly boosted commitment. When employees invest in the organization, they instigate identities to fulfill that task. Therefore, we hypothesize the following hypothesis:

***H4.*** *There is a positive association between employee investment and identity orientation.*

### Sequential Mediation of Thriving at Work and Employee Investment

Drawing on the overarching framework of the broaden–and–build theory, we argue that coping with challenge stressors serves to fulfill an individual’s need for competence (Ryan and Deci, 2000). This fulfillment produces positive emotions that motivate employees to break out of their comfort zone and learn and grow out of their self-imposed boundaries (Prem et al., 2017). As a result, they become committed (Porath et al., 2012) and tend to invest their personal resources in the job. As these employees are aligning their values and goals with organizational values and goals, they identify themselves with the organization. We, therefore, present the following hypothesis:

***H5.*** *Thriving at work and employee investment sequentially mediate the relationship between challenge stressors and identity orientation.*

**FIGURE 1:**
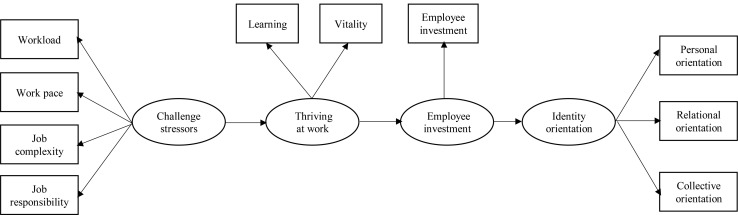
Conceptual framework.

The conceptual framework is presented in Figure 1.

## Methods

### Sample and Procedure

Data were collected from full-time employees of the banking sector. It included both commercial and corporate branches located in Pakistan. Only full-scale commercial banks’ employees were recruited for the study; employees from microfinance banks and other non-banking financial institutions were not contacted for this study. These banks had a variety of customers like big corporate clients, commercial accounts, SMEs, consumers, government and private institutions, etc.

Surveys were administered to the employees in the English language. English language is the official language of communication in Pakistan and is used as a medium of communication in banks. Therefore, it was not deemed necessary to translate the questionnaires into the native language of Pakistan. The cover letter in the questionnaire explained the purpose of the study and assured complete anonymity of respondents. A thorough explanation of the purpose and procedure of the study was offered to the participants. It was explained that participation was voluntary and that they could withdraw their participation or refuse to participate at any time without any penalty. The anonymity of the subjects was ensured. Written informed consent was obtained from the participants before participation in the survey.

A time-lagged research design was employed for data collection. To match the respondents of time 1 and time 2, in time 1 the respondents were asked to write the last four digits of their mobile phone number. The lead author explained the reason to them that in time 2 after 4 weeks, the respondents will be revisited for additional questions. This technique confirmed the anonymity of the respondents. At time 1, 370 questionnaires were distributed. The respondents answered about the demographics and rated challenge stressors and thriving at work. Two hundred ninety employees returned the complete filled-in questionnaires. Four weeks after the first wave of data collection, these 290 employees were contacted for the second wave of data collection. At time 2, the respondents were asked to provide the responses regarding employee investment and identity orientation. Two hundred twenty-five employees turned in completed surveys at the second phase of the data collection. Thus, 225 matched responses were obtained yielding a response rate of 60.81%. The majority of the respondents were male (79.10%). A total of 39.10% (88) of the respondents were in the 18–30 years old age group; 45.80% (103) of the respondents were 31–40 years old; 10.70% (24) employees belonged to the 41–50 age group; and 4.40% (10) of respondents were above 50 years of age. Data were collected from the top, middle, and lower management positions. Most of the respondents were married (68.90%) and a majority of them held a master’s degree (64%). The demographic information on the respondents is given in Table 1.

**TABLE 1:** Demographic information on the respondents.

	*n*	Percentage
**Gender**		
Male	178	79.10
Female	47	20.90
**Age**		
18–30 years	88	39.10
30–40 years	103	45.80
40–50 years	24	10.70
Above 50 years	10	4.40
**Marital Status**		
Single	70	31.10
Married	155	68.90
**Education**		
Bachelor’s degree	58	25.80
Masters	144	64.00
M.Phil	23	10.20
Total	225	

### Measures

#### Challenge Stressors

Challenge stressors are perceived as a source of enhancing mastery and personal growth. The 20-items scale was taken from Cavanaugh et al. (2000) study. The participants were asked to respond on a 5-point Likert scale ranging from “1 = strongly disagree to 5 = strongly agree” the extent to which events resulted in stress in their job. A sample item for workload is “I have to complete a great deal of work on this job;” for work pace is “To complete my work on time, I must work quickly;” for job complexity is “Tasks on my job use a variety of different skills and abilities;” and for job responsibility is “My job requires me to be accountable for my work and the work of others.” Cronbach’s alpha values were 0.900, 0.830, 0.895, and 0.866 of its four dimensions of workload, work pace, job complexity, and job responsibility, respectively.

#### Thriving at Work

Porath et al. (2012) established a 10-item scale for measuring thriving at work. This measure includes five items for learning and five items for vitality. For learning, a sample item is “I continue to learn more and more as time goes by;” and for vitality is “I feel alive and vital.” The reliability alphas were 0.684 and 0.607 of its dimension of learning and vitality, respectively. All the items were measured on a 5-point Likert scale ranging from “1 = strongly disagree to 5 = strongly agree.”

#### Employee Investment

We used a 3-item scale developed by Rusbult and Farrell (1983) to measure employee investment. The first item of the scale “In general, how much have you invested in your present job at your organization?” was measured by a 5-point Likert scale ranging from 1 (nothing) to 5 (a great deal). Next item “All things considered, are there issues uniquely associated with your present job at your organization that you would lose if you were to leave your present job?” was also measured on a 5-point Likert scale ranging from 1 (None) to 5 (a great many). The last item “How does your investment in your present job at your organization compare to what you think most people have invested in their jobs?” was measured on 5-point Likert scale ranging from 1 (I have invested less than most people) to 5 (I have invested more than most people). The Alpha coefficient for this scale was 0.630.

#### Identity Orientation

Based on the literature (e.g., Triandis, 1994; Brewer and Gardner, 1996; Cross and Vandiver, 2001) and the conceptualizations of three levels of identity given in the introduction, identity orientation was measured in its three distinct dimensions. Identity Orientation Scale developed by Vos and van der Zee (2011) was used to measure three levels of identity with 21 items. Personal identity orientation defines the personal behaviors and features and the sensation of individuality perceived by different persons. Sample items for personal identity orientation are: “I enjoy being different from others” and “It is important for me to do my own thing.” Relational identity orientation refers to a person’s notion with respect to relatedness to other individuals, i.e., individuals’ associations to others are their rational interpretations and their focus on maintaining relationships with others. The sample items include: “I enjoy maintaining personal relationships with others” and “I think that close others have much influence on my identity.” Collective identity orientation refers to self-conception in terms of group memberships. Sample items are: “I like to describe myself as a member of the groups to which I belong” and “It is very important to my identity to belong to a group.” All items were measured on a 5-point Likert scale ranging from “1 = strongly disagree to 5 = strongly agree.” Cronbach’s alpha reliabilities for the personal identity orientation, relational identity orientation, and collective identity orientation were 0.902, 0.918, and 0.943, respectively.

### Analytical Technique

To analyze the simultaneous impact of latent variables on each other, we used latent variable structural equation modeling (LV-SEM). In our model, we used some higher-order constructs (HOC), i.e., challenge stressors, thriving at work, and identity orientation. Challenge stressors were measured using four dimensions: workload, work pace, job complexity, and job responsibility. Thriving at work was measured by its two dimensions, i.e., learning and vitality. Identity orientation was measured by its three dimensions: personal orientation, relational orientation, and collective orientation. The use of LV-SEM was imperative in order to analyze the impact of HOCs while taking into account their underlying dimensions.

### Common Method Bias

Because of the fact that the data were collected from a single source, i.e., the individual employees, we introduced several procedural remedies to mitigate the threat of common method bias (CMB). First of all, we used a time-lagged research design that serves to create a temporal separation that hinders the respondents from eliciting the responses using the contextual cues for retrieval of information from long-term memory (Podsakoff et al., 2003). Thus, we collected the data for challenge stressors and thriving at T1; and at T2, 4 weeks after T1, data were collected for employee investment and identity orientation. Second, it was clearly communicated to the respondents that there are no right or wrong answers to the questions and that they should answer the questions with as much honesty as possible. Moreover, the anonymity of the respondents was ensured. These steps ought to serve the purpose of minimizing the social desirability bias. Furthermore, some reverse-coded questions were included. These steps further curtailed the happening of CMB. Additionally, we ran Harman’s single factor test to rule out the occurrence of CMB statistically. The single factor explained only 40.27% of the variance which is far less than the threshold of 50% (Podsakoff et al., 2003).

## Results

### Descriptive Statistics and Correlation

Table 2 represents the means, standard deviations, reliability coefficients, and correlation coefficients among the study variables. The correlations provided preliminary support for our hypotheses. Dimensions of challenge stressors were positively correlated with dimensions of identity orientation as well as with learning and vitality (dimensions of thriving at work), thus supporting H1 and H2. Learning was positively correlated with employee investment, while vitality did not have a significant correlation with employee investment. H3, therefore, was partially supported. Employee investment had positive correlations with dimensions of identity orientation, lending support to H4.

**TABLE 2:** Means, standard deviations, reliabilities, and correlations.

		Mean	SD	1	2	3	4	5	6	7	8	9	10
1	Workload	4.040	0.664	(0.900)									
2	Work pace	4.182	0.633	0.816**	(0.830)								
3	Job complexity	4.106	0.655	0.820**	0.734**	(0.895)							
4	Job responsibility	4.076	0.687	0.847**	0.743**	0.803**	(0.866)						
5	Learning	3.553	0.463	0.481**	0.534**	0.411**	0.491**	(0.684)					
6	Vitality	3.216	0.383	0.300**	0.407**	0.261**	0.261**	0.440**	(0.607)				
7	Employee investment	3.966	0.699	0.454**	0.306**	0.439**	0.433**	0.147*	–0.024	(0.630)			
8	Personal identity	4.071	0.681	0.455**	0.365**	0.484**	0.441**	0.180**	0.163*	0.399**	(0.902)		
9	Relational identity	4.061	0.626	0.650**	0.647**	0.607**	0.614**	0.541**	0.308**	0.504**	0.592**	(0.918)	
10	Collective identity	3.942	0.713	0.596**	0.565**	0.528**	0.576**	0.480**	0.152*	0.619**	0.532**	0.855**	(0.943)

N = 225. Values at diagonals in parentheses represent Cronbach’s alpha; while below the diagonal the estimated correlations are reported. ^∗^p < 0.05, **p < 0.01.

### Structural Model

#### Model Fit

Next, the structural model was analyzed to test the hypotheses using LV-SEM. The model indicated an excellent fit to the data. Chi-square value was 35.926 (*p* = 0.2896), which was insignificant showing an ideal model fit (Kenny, 2015). The values of goodness of fit index (GFI), comparative fit index (CFI), normed fit index (NFI), and Tucker-Lewis index (TLI) were 0.966, 0.997, 0.970, and 0.995, respectively. These values show an excellent fit (Diamantopoulos and Siguaw, 2000; Kline, 2015). The value of root mean square error of approximation (RMSEA) was 0.0234 which shows an excellent fit as described by MacCallum et al. (1996). The value of standardized root mean square residual (SRMR) was 0.0431 showing a good fit (Diamantopoulos and Siguaw, 2000). To assess the overall fit of the model, we used Hu and Bentler (1999) approach which proposes that fit of a model is considered good if either of the following two fit index combinations is fulfilled: CFI ≥ 0.95 and SRMR ≤ 0.09 or SRMR ≤ 0.09 and RMSEA ≤ 0.06. It is evident that our model fulfills both of these combinations, thus the fit of the model was excellent (Hu and Bentler, 1999).

#### Parameter Estimates

**FIGURE 2:**
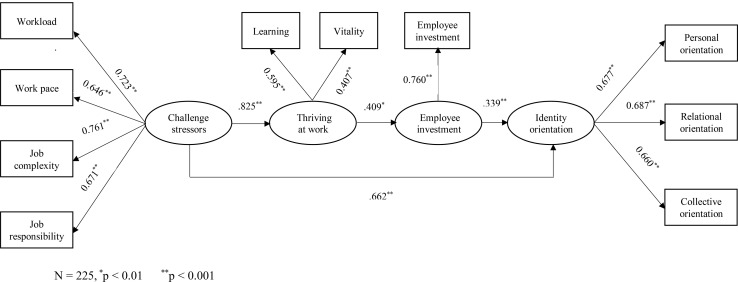
Path estimates. *N* = 225, ^∗^*p* < 0.01, ^∗∗^*p* < 0.001.

The results of hypotheses testing are reported in Table 3. H1 proposed a positive relationship between challenge stressors and identity orientation. The SEM modeling showed a strong positive path estimate from challenge stressors to identity orientation (0.662, *p* < 0.01). Thus, H1 was supported. H2 predicted a positive relationship between challenge stressors and thriving at work. Results showed that challenge stressors had a positive and significant effect on thriving at work (0.825, *p* < 0.01). H3 predicted a positive relationship between thriving at work and employee investment, and this hypothesis was supported as this effect was positive and significant (0.409, *p* < 0.01). In H4, we hypothesized a positive association between employee investment and identity orientation. This effect was also positive and significant (0.339, *p* < 0.01); thus, H4 was supported. The overall results of the measurement model and structural model are reported in Figure 2.

**TABLE 3:** Parameters estimates.

Path from	To	Parameter estimate	SE	*t*-Value
Challenge stressors	Thriving at work	0.825**	0.049	4.674
Thriving at work	Employee investment	0.409*	0.748	3.181
Employee investment	Identity orientation	0.339**	0.053	3.586
Challenge stressors	Identity orientation	0.662**	0.091	6.584

N = 225. *p < 0.01, **p < 0.001.

All the constructs in our model, except employee investment, are HOC which are being measured by multiple dimensions. As presented in Figure 2, all the factor loadings between HOCs and their respective lower-order dimensions (LOC) were significant, which indicates that underlying dimensions provide a reliable measurement of the constructs (Diamantopoulos and Siguaw, 2000).

### Mediation Analysis

H5 proposed sequential mediation of thriving at work and employee investment between the relationship of challenge stressors and identity orientation. The indirect effect for a sequential mediation can be obtained by multiplying *path a* (path from IV to MV1), *path d* (path from MV1 to MV2), and *path b* (path from MV2 to DV). In this way, path value for the indirect effect was 0.115 (0.825 × 0.409 × 0.339 = 0.115, *p* < 0.01). Table 4 shows the direct and indirect effects of challenge stressors on identity orientation. Findings revealed that the effect of challenge stressors on identity orientation was sequentially mediated by thriving at work and employee investment. H5 was, thus, supported. Moreover, the direct effect of challenge stressors on identity orientation (path c′) was positive and significant (0.662, *p* < 0.001), and so was the total effect (0.777, *p* < 0.001).

**TABLE 4:** Total, direct, and indirect effects of challenge stressors on employees’ identity orientation.

Effect	Product of coefficients	SE	*t*-Value
**Indirect effect**			
Challenge stressors → thriving at work → employee investment → identity orientation	0.825 × 0.409 × 0.339 = 0.115*	0.038	2.722
**Direct effect**			
Direct effect of challenge stressors on identity orientation (c′)	0.662**	0.091	6.584
**Total effect**			
Total effect of challenge stressors on identity orientation (c)	0.777**	0.094	7.474
**Squared multiple correlation**			
Thriving at work			0.681
Employee investment			0.168
Identity orientation			0.705

N = 225. *p < 0.01, **p < 0.001.

### Squared Multiple Correlations

The values of squared multiple correlations for all the endogenous variables are also given in Table 3. These values are analogous to R^2^ in multiple regression and indicate the amount of variance that independent variable(s) explain in the dependent variable. The values of squared multiple correlations for endogenous variables in our model ranged from 0.166 to 0.579 indicating that a reasonable amount of variance is being explained in the dependent variable by the independent variable(s) (Diamantopoulos and Siguaw, 2000).

## Discussion

The identity concept, although extensively applied, still remains a challenge for scholars to describe its related characteristics. Identity is a multidimensional construct. It monitors life pathways of individuals’ motivational directions, self- incorporation, and resolutions. It permits people to appeal strength from membership with social crowds and cooperatives (Podsakoff, 2007; Dutton et al., 2010). The current investigation was aimed at testing a model of relationship with five major hypothetical paths between study variables in direct and indirect relationships. The projected paths were found to be significant as challenge stressors was positively related to identity orientations. The findings also endorsed the mediating effects of thriving at work and employee investment in the relationship of challenge stressors with three dimensions of identity orientation, i.e., personal, relational, and collective orientation and showed the significant path estimates between them.

We concentrated on the challenge stressors, which create elite perceptions about workplace identity and employee identification. In stressful situations, employees feel depressed and annoyed when they are unable to handle occupational tasks (Rössler, 2012). The flip side of the identification was that when a job, a task or work is at risk, as in the cases when stressful conditions were regularly faced or when the identities existence of the work is in question, reactions might be predominantly negative (Widmer et al., 2012). These negative feedbacks may have a broader influence on identity and self-esteem (Ashforth and Mael, 1989; Sluss and Ashforth, 2007), and may lead to hospitalization and self-harm (Eliason and Storrie, 2009), exhaustion, post-traumatic stress syndromes, and even madness (Kleespies, 1993).

According to hypothesis 1, which depicted the positive association between challenge stressors and identity orientation of employees, it is noted that the feeling of shared social identity amplified among members if they take stress as a challenge. The key objective of this research was to explore the link between challenge stressors and employees’ identity orientation with the help of the broaden–and–build theory. The results depicted a positive relationship between these variables. These results could be explained with the help of the broaden-and-build theory. Positive emotions are generated upon successfully tackling challenging job tasks. These positive emotions are personal resources that are valuable in their own right. These resources help to enhance their commitment and fit with the job and the organization. Hypothesis 2 depicted the positive association of challenge stressors with thriving at work which was supported by the results. These results are consistent with the work of Prem et al. (2017) which linked challenge stressors with learning and vitality through the mediating role of cognitive appraisal. Positive emotions foster the learning of the employees and they work with elevated passion and vigor.

Similarly, hypothesis 3 explained the positive association between thriving at work and employee investment. When workers feel passionate and motivated at the workplace, they take ownership of their work and invest their skills and potential for the progress of the organization. van der Walt (2018), in their study, pointed out the relation of workplace spatiality, thriving at work and employee engagement. We argue that if employees engage in the organization, then they invest their efforts and skills for completion of the task. In hypothesis 4, employee investment was linked with identity orientation. This hypothesis was supported. Employees who are more committed, engaged and invested in their work role draw more satisfaction from their job and more recognition at workplace (Rothbard and Edwards, 2003; He et al., 2014). Hypothesis 5 predicted the mediating mechanisms of thriving at work and employee investment. Our results also supported this hypothesis by showing a positive and significant path estimate.

### Theoretical Implications

The current study investigated the effect of challenge stressors on identity orientation of the employees. Prior literature has demonstrated the detrimental effects of identity threats due to perceived stress in organizations (Berjot et al., 2013). Wegge et al. (2012) propose that organizational identification can function as a valuable resource in coping with stressors. Willis et al. (2019) explored the link between social identification and stress. It is important to recognize the role of challenge stressors in the growth of identity. This study contributes to the literature on three different levels. First, drawing on the broaden–and–build theory, this study empirically established challenge stressors as an antecedent of employees’ identity orientation. This is a novel contribution as this is one of the first studies which theorized and empirically established this link.

Moreover, theoretical contributions of the current study go beyond as we have framed our research questions under the overarching framework of the broaden–and–build theory. In contrast, prior stress research has mostly relied on the transactional theory of stress and coping. Our findings advocate different lines of consideration as paradigms modified to accept stress as challenge and manage the talent. Second, this study attempted to explore the mediating role of thriving at work and employee investment in the relationship between challenge stressors and identity orientation. Our results suggested that these variables sequentially mediated this relationship. This is also a unique contribution as according to the best of our knowledge, no study in the literature has tested the mediating role of thriving at work and employee investment between challenge stressors and employees’ identity orientation. Third, this study was conducted in a developing South Asian country, i.e., Pakistan. According to cultural studies done by Hofstede (1983), Pakistan is in sheer contrast to the developing countries on a number of cultural factors. Thus, this study offers unique contributions in a collectivistic culture. Pakistan is a country with collectivistic culture, therefore, in this culture, it is important to be a member of a group. Thus, collective identity orientation is more important in this collectivistic society as belonging to a group brings more benefits and group membership provides a sense of security to the individuals.

### Practical Implications

The findings suggest that organizations interested in addressing job-related stress to retain and manage talent and reduce employee turnover need to, and should, focus on encouraging challenge-related stress. Training and development plans ought to be designed for the employees, which focus on taking the stress positively. These training programs would improve the skills and practices of employees, thus encouraging them to focus on their self-actualization and self-esteem. The employees might be encouraged to engage in job crafting. Job crafting encourages individuals to redesign and personalize the aspects of their job as it fosters engagement, job satisfaction, resilience, and thriving (Wrzesniewski and Dutton, 2001).

As talent management is a contemporary issue for the management, with the help of this study, the managers could monitor their employees and encourage the interpersonal, relational, and collective interaction among employees. Intrinsic and extrinsic rewards play a vital role in motivating employees. Intrinsic rewards and recognition must be extended to the employees who achieve challenging goals so that these employees could treat hard-to-achieve goals as challenging rather than as a burden.

### Limitation and Suggestions for Future Research

Apart from several strengths and contributions, there are certain limitations of this study that arise from methodological as well as theoretical issues. First, as we relied on single-source responses, CMB might become a potential threat. Although we employed a time-lagged research design and took several procedural and statistical measures to minimize CMB, we advise future researchers to employ a longitudinal and/or multi-source research design to avoid CMB and single-source bias. Another limitation is relying only on banking sector employees for data collection. This limits the generalizability of the findings. Future research might include numerous industrial sectors to make the findings more generalizable.

On the theoretical front, with regards to identity orientation conceptualization, future research will need to determine whether there are, in fact, three different loci of self-definition and whether these correspond to particular social motivations, relevant self-knowledge, and frames of reference for evaluation. Research on identity orientation is a promising area for research, as there is scarce research in this area. This study has explored the impact of challenge stressors on identity orientation through the mediating roles of thriving at work and employee investment. This relationship needs to be explored through other mediators too. Boundary conditions should be taken into account; in this connection, leader roles and HR practices could be checked as moderators to set the context in which challenge stressors impact on identity orientation.

## Data Availability Statement

The raw data supporting the conclusions of this article will be made available by the authors, without undue reservation.

## Ethics Statement

The studies involving human participants were reviewed and approved by the ethics committee of Government College University Faisalabad, Pakistan. The patients/participants provided their written informed consent to participate in this study.

## Author Contributions

SS, SH, BL, UI and IS contributed to the definition of research objectives, model and hypotheses, data analysis plan, and approval of the final manuscript. SH and BL contributed to the provision of materials (i.e., questionnaires). SS and SH participated in data collection. SS, BL, and UI participated in data analysis. SS and SH participated in writing the main article. SS and IS contributed to article revision and proofreading. All authors contributed to the article and approved the submitted version.

## Conflict of Interest

The authors declare that the research was conducted in the absence of any commercial or financial relationships that could be construed as a potential conflict of interest.
